# Supplementary material for: Assessment of Anti-Human Leukocyte Antigen (HLA)-Antibody-Dependent Humoral Response in Patients before and after Lung Transplantation
Source: Medicina (Kaunas). 2022 Nov 30;58(12):1771. doi: 10.3390/medicina58121771 (PMC9781897; doi:10.3390/medicina58121771)
Supplement: Supplementary file 1 [file medicina-58-01771-s001.zip › medicina-1961598-supplementary.pdf]

Supplementary materialas

Table S1. Descriptive statistics without and with division into groups according to the disease

Variables/group		N	mean	SD	median	Q_1	Q_3	x_{min}	x_{max}
**Age [years]**		152	38.4	15.4	37.0	24.0	52.5	10.0	70.0
**Age [years]**	**CF**	62	26.2	9.0	24.0	20.0	32.0	13.0	54.0
	**COPD**	36	51.8	9.9	55.0	46.5	58.5	24.0	70.0
	**ILD**	41	47.2	12.2	44.0	39.0	59.0	20.0	68.0
	**IPAH**	13	31.5	13.0	34.0	20.0	39.0	10.0	54.0
**PRA [%%]**		145	3.2	7.9	0.9	0.5	2.8	0.1	66.4
**PRA [%%]**	**CF**	61	2.4	3.5	0.9	0.6	2.5	0.2	17.1
	**COPD**	33	2.0	2.7	0.6	0.4	2.6	0.2	11.0
	**ILD**	39	4.0	9.9	1.2	0.7	3.0	0.1	57.9
	**IPAH**	12	8.1	18.9	1.3	0.5	3.5	0.3	66.4

Legend: CF – Cystic Fibrosis, COPD – …….., ILD – …….., IPAH – ……., SD – standard deviation, Q_1 – lower quartile, Q_3 – upper quartile
